# Perception of the Professional Knowledge of and Education on the Medical Technology Products among the Pharmacists in the Baltic and Nordic Countries—A Cross-Sectional Exploratory Study

**DOI:** 10.3390/pharmacy4040029

**Published:** 2016-10-13

**Authors:** Daisy Volmer, Aleksandra Sokirskaja, Raisa Laaksonen, Kirsti Vainio, Niklas Sandler, Kjell H. Halvorsen, Reidun Lisbet Skeide Kjome, Sveinbjörn Gizurarson, Ruta Muceniece, Baiba Maurina, Jurgita Dauksiene, Lilian Ruuben, Ingunn Björnsdottir, Tagne Ratassepp, Jyrki Heinämäki

**Affiliations:** 1 Institute of Pharmacy, Faculty of Medicine, University of Tartu, 1 Nooruse Str., 50411 Tartu, Estonia; sokirskaja@hot.ee (A.S.); jyrki.heinamaki@ut.ee (J.H.); 2 Division of Pharmacology and Pharmacotherapy, Faculty of Pharmacy, University of Helsinki, Viikinkaari 9 (P.O. Box 56), 00014 Helsinki, Finland; raisa.laaksonen@helsinki.fi; 3 School of Pharmacy, University of Eastern Finland, P.O. Box 1627, 70211 Kuopio, Finland; kirsti.vainio@uef.fi; 4 Pharmaceutical Sciences, Åbo Akademi University, BioCity, Artillerigatan 6A FI, 20520 Turku, Finland; niklas.sandler@abo.fi; 5 Department of Pharmacy, Faculty of Health Science, UiT The Arctic University of Norway, 9037 Tromsø, Norway; kjell.h.halvorsen@uit.no; 6 Department of Global Public Health and Primary Care, University of Bergen, 5018 Bergen, Norway; reidun.kjome@isf.uib.no; 7 Faculty of Pharmaceutical Sciences, University of Iceland, 107 Reykjavik, Iceland; sveinbj@hi.is; 8 Department of Pharmacy, Faculty of Medicine, University of Latvia, LV-1050 Riga, Latvia; ruta.muceniece@lu.lv; 9 Faculty of Pharmacy, Riga Stradins University, LV-1007 Riga, Latvia; Baiba.Maurina@rsu.lv; 10 Department of Drug Technology and Social Pharmacy, Faculty of Pharmacy, Medical Academy, Lithuanian University of Health Sciences, 44307 Kaunas, Lithuania; jurgita.dauksiene@gmail.com; 11 Tallinn Health Care College, 13418 Tallinn, Estonia; lilian.ruuben@ttk.ee; 12 School of Pharmacy, Faculty of Mathematics and Natural Sciences, University of Oslo, 0316 Oslo, Norway; ingunn.bjornsdottir@gmail.com; 13 Medical Device Department, Estonian Health Board, 50303 Tartu, Estonia; Tagne.Ratassepp@terviseamet.ee

**Keywords:** medical technology, education, pharmacy students, practicing pharmacists, professional competency

## Abstract

With increased development of medical technology (MT), new challenges emerge related to education and training of pharmacists and other healthcare specialists. Currently, only a few universities in the EU promote MT education and research. Objectives: The aim of this study was to evaluate the current status, views on, and need for the education on MT for the pharmacy students and practicing pharmacists in the Baltic and Nordic countries. Methods: The representatives of higher education institutions and community/hospital pharmacists from six Baltic and Nordic countries participated in a qualitative cross-sectional exploratory internet-based study from May to October 2014. Results: Approximately two-third of the respondents considered professional knowledge about MT products important for pharmacists, but half of them had never participated in any MT courses. More practicing pharmacists than representatives of academia underlined the need for increased MT education for pharmacy students in the future. Conclusions: The pharmacists in the Baltic and Nordic countries consider the professional knowledge about MT as pertinent in their education and work. The limited number and status of MT courses available today, however, is a major concern among both pharmacy students and practicing pharmacists in these countries. In the future, increasing education combining theory and practice about MT products would be one possible solution to overcome this challenge.

## 1. Introduction

Medical technology (MT) encompasses a wide range of health care products and is used to diagnose, monitor or treat diseases or conditions affecting humans. MT covers medical devices (MDs), drug delivery products (DDPs, medicinal products integrated with device), procedures and organizational systems used in health care [1].

While there are many potential therapeutic benefits in using MT, this relies on the correct use of the equipment, and misuse may compromise patient safety. A UK patient safety audit found that nearly 10% of safety incidents reported from intensive care and high dependency units involved MDs [2].

In the Baltic and Nordic countries, community and hospital pharmacies are an important source for counselling and dispensing of MDs and DDPs. Some alternative channels for purchasing personal MDs include e.g., supermarkets, opticians and regular shops for patches and patient care products, and special shops for blood pressure monitors and other MT products. Increasingly, people prefer to buy MDs from pharmacies as they are considered to be preparations closely related to pharmaceutical products [3,4]. Today, community pharmacies are also a legal distribution source of MT products. With the implementation of Directive 2011/24/EU on the application of patients' rights in cross-border health care, community pharmacies are responsible for providing counselling and assuring the safe and appropriate use of MT products in the same way as for medicinal products [5]. Previous studies suggest that the proper use of MDs and DDPs can influence medication adherence and illness control [6]. Pharmacists are ideally positioned to educate patients, who use medications associated with MDs and DDPs [7,8,9,10,11].

With the rapid development of MT, new challenges occur related to education and training of pharmacists and other healthcare specialists. At present, only few universities promote MT in the EU through offering postgraduate education and research projects [12,13]. For example, in the UK, the Sheffield Hallam University in collaboration with the NSF Health Sciences Medical Devices offers international quality and regulatory training courses, as well as professional qualifications on MD [14]. In the USA, the significance and need for this kind of education have been better recognized [15,16]. The lack of high-level education and training in MT is a major concern since in the future the number of different medical, biomedical and pharmaceutical services will steadily increase in health care. Pharmacists will have an important role in delivering and counselling on MT products for patients.

The aim of this cross-sectional exploratory study was to evaluate the current status, views on, and need for, education provided on MT to pharmacy students and practicing pharmacists in the Baltic and Nordic countries.

## 2. Materials and Methods

### 2.1. Study Design and Sampling

Qualitative electronic survey using a web platform *eFormular* was employed as a research tool for data collection in this cross-sectional study. In the Baltic and Nordic countries, all parties involved with MT education and practice in pharmacy were asked to participate.

The invitation to participate was forwarded to all higher education institutions (HEI) providing the under- and postgraduate training of pharmacists (MSc Pharm) and assistant pharmacists (BSc Pharm) as well to the professional organizations of community and hospital pharmacists (representing practicing pharmacists). All three Baltic countries—Estonia, Latvia and Lithuania—participated in the survey. Finland, Iceland and Norway participated from the Nordic countries, while Denmark and Sweden declined the invitation (Table 1). In all countries the data collection was conducted according to the general ethical principles of biomedical research. Separate approval from ethics committee was not required for this type of study (confirmed by the Research Ethics Committee of the University of Tartu).

### 2.2. Survey Instrument

The initial ideas regarding the necessity of professional knowledge of MDs and DDPs for pharmacists were obtained from an earlier national study in Estonia [3,4]. The initial survey instrument was developed by the panel of researchers at schools of pharmacy in the participating countries. The specialists in MT (Estonian Health Board) and health statistics as well as the representatives of community pharmacies (*n* = 5) were consulted to ensure the content and face validity of the survey instruments. In the later stage, it was decided to design two separate survey instruments. The first one was intended for the representatives of academia (teaching staff and students) and the second one for the professional organizations with some changes in wording. The content of these two survey instruments, however, was identical. The survey instruments consisted of total 9 multiple choice and 4 non-multiple choice questions on the following topics:

- the importance of professional knowledge about MDs for pharmacists;
- the courses available focusing on MDs and DDPs at universities and for practicing pharmacists;
- the background of specialists providing training about MDs and DDPs at universities and for practicing pharmacists;
- the future education need about MDs and DDPs for pharmacy students and pharmacists.

The survey instruments are provided as “supplementary materials”.

### 2.3. Data Collection

The survey instrument was asked to be completed by academic staff members working with the development and implementation of pharmacy curriculum as well as by BSc Pharm/MSc Pharm students at the HEIs. One jointly completed survey instrument from the academic staff members of each higher education institution was expected to be received. One to two completed joint survey instruments were asked from the pharmacy students (one from those universities providing only MSc degree education and two from those giving both BSc and MSc degree education in pharmacy). Professional organizations received the survey instrument via the university contacts with a request to complete only one joint survey instrument by representatives of community and hospital pharmacies, respectively. As shown in Table 1, several completed survey instruments were received from some participating countries.

### 2.4. Data Analysis

The data were collected onto *eFormular*, coded and analyzed by using MS Excel version 14.0. For the open questions, principles of qualitative content analyses were used [17]. The results have been grouped in representatives of academia (teaching staff and pharmacy students), and in representatives of professional associations (community pharmacy and hospital pharmacy) and practicing pharmacists. Grouping by countries was not used due to uneven representation of the countries in the survey.

## 3. Results

A total of 50 responses were obtained and analyzed in the survey. All HEIs providing pharmacy education in the participating countries were represented in the study. Of the professional organizations or practicing specialists we received at least one joint reply from the community and hospital pharmacy, except hospital pharmacists from Finland (Table 1). Total 34 respondents were from the academia (14 teaching staff members and 20 BSc and MSc pharmacy students), and 16 respondents were from the professional organizations and practicing specialists (11 from the community pharmacy and 5 from the hospital pharmacy).

According to the respondents, there is a lack of available MT product courses across the Baltic and Nordic countries. The minor variation in responses between the survey participating countries could be explained by the differences in the education and practice systems in these countries. About two-third of the respondents strongly agreed with the claim that professional knowledge about MT products is important for pharmacists and increases the quality of community pharmacy services. About two-third of the respondents strongly agreed with the claim that professional knowledge about MT products is important for pharmacists and increases the quality of community pharmacy services.

### 3.1. Perceptions of the Current Status of the Courses Available about MT Products

The availability of lectures and courses on MT products for pharmacy students and practicing pharmacists, according to the representatives of academia, practicing pharmacists and professional associations, in the Nordic and Baltic countries is presented in Table 2.

About one fifth of the representatives of professional organizations reported that continuing education courses, focusing on different aspects of MT products, are available for practicing pharmacists. Based on the responses of the academia group, teachers seemed more likely than students to agree that theoretical and practical MT courses are available and more students agree on availability of theoretical than practical courses about MT products. A short description of compulsory courses related to MT products in the study countries is presented in the additional information included to the article.

The representatives of practicing pharmacists including professional organizations described their views on the existing courses for practicing pharmacists on MT products. The *Finnish* respondents reported that there are no general courses available on MDs to date, but for example, in some asthma-related courses, training about the correct use of inhalation devices is given. Furthermore, some information about measuring blood pressure is presented in the courses on hypertension. The *Estonian* respondents described some specialized courses: practical instructions about the use of DDPs (i.e., insulin pens) and diagnostic medical devices (i.e., blood pressure monitors, glucometers).

The *Lithuanian* respondents described courses on general principles of MT products and the *Latvian* respondents courses on the use of glucometers. In both countries, there are also courses on asthma (including the correct use of inhalators) and on hypertension (including the correct measurement of blood pressure).

The *Icelandic* respondents reported that occasionally some medical device companies provide training events for practicing pharmacists. The respondents from *Norway* informed that no courses were available for practicing pharmacists, but at the universities they have a group of students, who invite industry or other pharmacy-related specialists or health care professionals to give lectures once or twice per term on a specific topic (i.e., an elective lecture entitled “Asthma inhalers, difficulties and solutions”, free of charge for pharmacists).

### 3.2 Perceived Professional Competency of Specialists Providing the Training about MT Products

About half of the practicing pharmacists in community pharmacies and one fifth of the practicing pharmacists in hospitals reported that the teachers in their continuing education courses were professionals in the field of MT products (Table 3). In addition, more than half of the representatives of hospital pharmacists reported that specialists from MT industry and practicing medical doctors and nurses were involved at the continuing education courses about MDs and DDPs However, over half of the representatives of community pharmacists named university lecturers without special education of MTs as common trainers about MT products. University lecturers with no specific education about MT were named by majority of academia group as frequent providers about information on MT products. Currently the HEIs in the participating countries do not employ specialists with specific education on MT. Specialists from other fields may have extended, practical, knowledge in the specific field of MT, i.e., about the use of blood pressure monitors, inhalators, glucometers, cholesterol meters, hemoglobin meters and insulin delivery devices.

### 3.3. Educational Perspectives on MT Products in the Future

The majority of the representatives of professional organizations and practicing pharmacists expressed the urgent need to increase the number of lectures and practical seminars about MT products for pharmacy students. In the academia group, about half of the respondents shared the same opinion (Table 4).

Academic and practicing specialists groups considered “teaching the teachers” type of (international) courses a good solution to increase pharmacy students’ and pharmacists’ professional competency on MT products. According to all respondents educational courses and seminars could be organized by regional institutions (universities, hospitals, pharmaceutical companies) and professional associations as well as European and international organizations, i.e., representative of medical technology industry in Europe—Eucomed.

## 4. Discussion

To the best of our knowledge, this is the first international study evaluating the existing status and need of MT education for pharmacy students and practicing pharmacists (including postgraduate education). Previous international research has revealed that the corresponding education is fragmented and may impede the professional competency of community and hospital pharmacists in counseling of the use of MDs and DDPs [3,4,6,7,8,9,10].

The results of current study support former international studies—there is a lack of available specific MT product courses across the Baltic and Nordic countries. Currently provided knowledge about MDs and DDPs for pharmacy students and practicing specialists in the Baltic and Nordic countries may differ to some extent, and be more theoretical for pharmacy students and covering mostly detailed practical aspects for pharmacists. For practicing pharmacists, the training is mostly given by the specialists from the MT industry, nurses and physicians. For pharmacy students, the education is mostly provided by the university teaching staff without special education in the field of MT. There is a good example from Norway where students have taken initiative to organize the lectures and courses by inviting educators from MT industry as well as practicing physicians and nurses.

Described trends could also be seen in the former MT education research, where continuing education courses oriented on the practical aspects of MT products, i.e., effective and safe use of DDPs have been mostly studied. Casset et al. (2014) investigated asthma management and inhalation techniques among community pharmacists in France and compared the results of 1999 and 2009 studies [18]. Within ten years, the health care authorities encouraged the involvement of community pharmacists in patient care and education in order to improve asthma control. However, knowledge of asthma management is still average, pointing to the need to join different counselling skills about disease, the use of medicines and medical devices [18]. Basheti et al. (2009) reported about the need for continuing education and practice with patient training in order be able better maintain their inhaler technique demonstration skills [10].

On the other hand, few countries in Europe emphasize the need for theoretical knowledge in MT. For example, some pharmacy schools in Bulgaria provide separate courses in MDs within the MSc (Pharm) curriculum in the 5th semester containing total 30 h of studies [19]. According to the authors, it has been considered to establish unified state requirements for the present courses in MT as there is an increased demand for this type of professional knowledge among practicing pharmacists [19]. In addition, Sakuma (2013) has emphasized that teaching the fundamental framework of MD regulations and standards is more important than teaching only detailed guidelines for specific MDs [20]. The authors recommended education through case studies where students can analyze examples of specific MDs, i.e., approval process and use, as an example of effective methods. These trainings could be implemented together with research activities conducted by students [20]. In the future planning of MT education combined approach of theoretical knowledge and practical skills could be suggested in undergraduate as well as continuing pharmacy education.

This study presented considerable difference in opinions of different respondent groups about increasing the education of MT products for pharmacy students in the future. While academic staff and pharmacy students found this knowledge less important, the representatives of professional organizations and practicing pharmacists would demand more training given during pharmacy studies about MDs and DDPs. In our previous study, only 15% of community pharmacists in Estonia indicated university education as relevant source of professional knowledge about MT products [4]. The difference in the opinions of respondents’ groups could be due to the statutory role of practicing pharmacists in assuring the quality and safety of dispensed MDs and DDPs, and due to their insufficient professional competency to complete this task. Respondents would value the possibility to have international courses about MT products and have listed universities, governmental institutions, international organizations and representatives of pharmaceutical industry as possible providers of educational courses about MDs.

Based on the results and feedback of this study, we plan to establish the Baltic-Nordic research cluster together with the leading public and private sector organizations acting in different areas of pharmacy and healthcare sectors. The main scope of the cluster is to gain understanding of the challenges related to education, implementation, application and regulation of modern MT innovations in the health care and society. This knowledge could be used in improving the quality and safety of MT product related interventions at primary care level and in improving the quality of education provided on this field for students and practicing specialists.

### Study Limitations

In this study, there was an uneven representation of participating countries and respondent groups. We received more responses from Estonia and Norway, compared to Iceland and Lithuania. Furthermore, the number of respondents from academia was higher compared to practicing specialists and representatives of the professional organizations. There were different views and practices about the MT products in the Baltic and Nordic countries, in addition did Sweden and Denmark decline the invitation, and thus the full overview of the current available MT education may not have been gained. In addition, the joint survey protocol used in this study can result in partially skewed information.

## 5. Conclusions

Based on this cross-sectional exploratory study, the pharmacists in the Baltic and Nordic countries consider the professional knowledge about MT as relevant in their education and work. However, the number of specific courses on MDs and DDPs for students and pharmacists are limited, or even missing in some of the participating Baltic and Nordic countries. Respondents agreed that the basic knowledge of MT products is a part of professional competency of practicing pharmacists. In the future, education combining theory and practice about MT products would be an interesting approach to improve MT knowledge in pharmacies.

## Figures and Tables

**Table 1:** The higher education institutions (HEIs) and professional organizations involved in the survey.

Country/Total Number of Respondents	HEI/Professional Organization and Respective Number of Responses
Estonia (*n* = 12)	University of Tartu (*n* = 3); Tallinn Health Care College (*n* = 2); Estonian Society of Hospital Pharmacists (*n* = 1); Estonian Pharmacists’ Association (*n* = 1); Estonian Pharmacies Association (*n* = 1); Estonian Pharmacy Society (*n* = 1); Pharmacy Chain Euroapteek (*n* = 1); Community Pharmacy Managers (*n* = 2)
Finland (*n* = 6)	University of Helsinki (*n* = 2); University of Eastern Finland (*n* = 2); Åbo Akademi University (*n* = 1); Association of Finnish Pharmacies (*n* = 1)
Iceland (*n* = 4)	University of Iceland (*n* = 2); The National University Hospital of Iceland (*n* = 1); Community Pharmacy Manager (*n* = 1)
Latvia (*n* = 9)	University of Latvia (*n* = 2); Stradins University (*n* = 5); Latvian Pharmacists’ Association (*n* = 2)
Lithuania (*n* = 4)	Lithuanian University of Health Sciences (*n* = 2); Lithuanian Pharmaceutical Society, Hospital Pharmacy Section (*n* = 1); Lithuanian Union of Pharmacists (*n* = 1)
Norway (*n* = 15)	University of Oslo (*n* = 1); University of Bergen (*n* = 2); UiT The Arctic University of Norway (*n* = 5); The Nord-Trøndelag University College (*n* = 5); The Norwegian Association of Pharmacists (*n* = 1); Norwegian Pharmaceutical Students’ Association (*n* = 1)

**Table 2:** The availability of specific training about MT products for pharmacy students and practicing pharmacists in the Nordic and Baltic countries as reported by survey respondents (*n* = 50).

	Students (*n* = 20)		Teaching Staff (*n* = 14)		Community Pharmacists (*n* = 11)		Hospital Pharmacists (*n* = 5)	
	YES	NO	YES	NO	YES	NO	YES	NO
General principles of MT products	17	3	10	4	3	8	1	4
Practical use of MDs	10	10	10	4	4	7	1	4
Practical use of DDPs	9	11	10	4	4	7	1	4

**Table 3:** Specialists providing the training on MT for pharmacy students and practicing pharmacists in the Nordic and Baltic countries by survey respondents (*n* = 50).

	Students (*n* = 20)		Teaching Staff (*n* = 14)		Community Pharmacists (*n* = 11)		Hospital Pharmacists (*n* = 5)	
	YES	NO	YES	NO	YES	NO	YES	NO
Specialists in the field of MT *	4	16	5	9	6	5	1	4
University teachers without specialization on MT	14	6	13	1	7	4	1	4
Representatives of MT industry	6	14	4	10	5	6	3	2
Practicing doctors or nurses	4	16	6	8	5	6	3	2

* MT = medical technology.

**Table 4:** Opinions about the need to increase the share of MT education for pharmacy students of survey participants by country (*n* = 50).

		Students (*n* = 20)	Teaching Staff (*n* = 14)	Community Pharmacists (*n* = 11)	Hospital Pharmacists (*n* = 5)
To a	great extent	4	2	5	3
	considerable extent	8	1	3	1
	moderate extent	5	7	3	1
	small extent	3	4	-	-

## Supplementary Materials

The following are available online at http://www.mdpi.com/2226-4787/4/4/29/s1, Table S1: Compulsory and elective courses about MT products in the Baltic and Nordic countries.
