# Supplementary material for: Perception of the Professional Knowledge of and Education on the Medical Technology Products among the Pharmacists in the Baltic and Nordic Countries—A Cross-Sectional Exploratory Study
Source: Pharmacy (Basel). 2016 Oct 13;4(4):29. doi: 10.3390/pharmacy4040029 (PMC5419374; doi:10.3390/pharmacy4040029)
Supplement: Supplementary file 1 [file pharmacy-04-00029-s001.zip › pharmacy-148992-supplementary/pharmacy-148992-supplementary1.docx]

Supplementary Materials: Perception of the Professional Knowledge and Education on the Medical Technology Products among the Pharmacists in the Baltic and Nordic Countries—A Cross-Sectional Exploratory Study

Daisy Volmer, Aleksandra Sokirskaja, Raisa Laaksonen, Kirsti Vainio, Niklas Sandler,
Kjell H. Halvorsen, Reidun Kjome, Sveinbjörn Gizurarson, Ruta Muceniece, Baiba Maurina, Jurgita Dauksiene, Lilian Ruuben, Ingunn Björnsdottir, Tagne Ratassepp and Jyrki Heinämäki

**Table S1.** Compulsory and elective courses about MT products in the Baltic and Nordic countries.

| **Country** | **Course description** | **Number of ECTS */h** |
| --- | --- | --- |
| Estonia |  |  |
|  | Compulsory courses: |  |
|  | Biophysics (4ECTS)—general working principles of MDs. | 5 h |
|  | Primary care medicine (5 ECTS)—practical introduction to how to use PPDs (inhalers, insulin pens). | 3 h |
|  | Social pharmacy and drug safety (11 ECTS)—practical introduction to how to use DDPs and diagnostic MDs. | 6 h |
|  | Traineeship at community and hospital pharmacy (37 ECTS)—practical intro to MDs used in hospital and ambulatory setting. | 6 h |
|  | Elective courses: |  |
|  | Medical devices at community pharmacy | 2 ECTS |
|  | Minimally invasive medical technology. | 2 ECTS |
|  | Spectacles, Contact Lenses and Refractive Surgery. | 1 ECTS |
|  |  | Total 20 h/50 h |
| Finland |  |  |
|  | Compulsory courses: |  |
|  | Pharmacotherapy | 8 h |
|  | Human physiology and anatomy | 5 h |
|  | Patient education and counselling 3 | 3 h |
|  | Pharmaceutical technology lectures and practicals | 2 h |
|  | Basics of phytotherapy | 1 h |
|  | Pharmacology and toxicology | 1 h |
|  | Drug discovery and development | 4 h |
|  | Medical devices | 2 h |
|  | In advanced studies and elective studies some individual lectures and practices. | 2 h |
|  | Traineeship at community pharmacy | 2 h |
|  | No information available about elective courses |  |
|  |  | Total: 30 h |
| Iceland |  |  |
|  | No information available. |  |
| Latvia |  |  |
|  | Pharmaceuticals | 4 h |
|  | Practical pharmacy | 6 h |
|  | First aid | 2 h |
|  | Clinical pharmacy | 4 h |
|  | Patophysiology | 4 h |
|  | Anatomy and physiology | 4 h |
|  | Information and consultations in pharmacy | 4 h |
|  | Design of new drug formulations | 6 h |
|  | Clinical pharmacology | 4 h |
|  | Healthcare goods | 48 h |
|  | No information available about elective courses | Total: 86 h |
| Lithuania |  |  |
|  | No specific courses available |  |
| Norway |  |  |
|  | Pharmacotherpy and clinical pharmacy | 8 h |
|  | Pharmaceutical technology | Number of h not determined |
|  |  |  |
|  |  |  |
|  | Pharmacology | 1 h |
|  | Sterile formulations | 2 h |
|  | Pharmaceutical practice | 3 h |
|  | Pharmacy traineeship | Number of h not determined |

* 1 ECTS elective courses—10 h auditory work and 10 h independent work.
