# Supplementary material for: Perception of the Professional Knowledge of and Education on the Medical Technology Products among the Pharmacists in the Baltic and Nordic Countries—A Cross-Sectional Exploratory Study
Source: Pharmacy (Basel). 2016 Oct 13;4(4):29. doi: 10.3390/pharmacy4040029 (PMC5419374; doi:10.3390/pharmacy4040029)
Supplement: Supplementary file 1 [file pharmacy-04-00029-s001.zip › pharmacy-148992-supplementary/pharmacy-148992-supplementary2.docx]

**Current Views and Future Perspectives of Medical Device Education** **for Pharmacy Students and Pharmacists in the Nordic and Baltic Countries**

Dear colleague,

The use of medical devices (MDs) and various drug-delivery products (DDPs) is rapidly growing in the health care. Community pharmacies are an important source for counselling and dispensing of MDs and DDPs.

**The aim of this study is to assess the current education of MDs and DDPs for pharmacy students and pharmacists in the Nordic and Baltic countries.**

For a better understanding of the topic, the following definitions describe some aspects relating to medical devices in more detail:

*Medical device (MD)—*any instrument, apparatus, appliance, material or other article, whether used alone or in combination, including the software necessary for its proper application, intended by the manufacturer to be used for human beings for the purpose of:

- diagnosis, prevention, monitoring, treatment or alleviation of disease;
- diagnosis, monitoring, treatment, alleviation of or compensation for an injury or handicap;
- investigation, replacement or modification of the anatomy or of a physiological process;
- control of conception; and which does not achieve its principal intended action in or on the human body by pharmacological, immunological or metabolic means, but which may be assisted in its function by such means.

*MDs can be further classified as:*

*Personal medical device (PMD)—*not defined in the European medical device legislation. PMDs, as a major sub-group of MDs, could be described as portable, consumer-focused technologies that can be used at home for health and fitness trending, chronic disease management, and elderly patient monitoring (examples of PMDs: blood pressure monitors, glucometers, cholesterol meters, pregnancy/ovulation tests).

Or:

*Drug-delivery product (DDP)*—a device that is intended to administer a medicinal product within the meaning of the medicinal product directive (examples of DDPs: inhalation devices, insulin pens).

The questionnaire is intended to be **jointly** completed by academic staff members dealing with the development and implementation of pharmacy curriculum. **One completed questionnaire is expected from each participating university.**

In addition, the views of BSc Pharm or MSc Pharm students (3rd or 4th year) are kindly asked to be collected. **One to two questionnaires** (one from those universities, providing only Bachelor or Master studies and two from those universities have both Bachelor and Master studies in pharmacy) are expected to **be filled in based on the joint opinions of pharmacy students.**

Before completion of the questionnaire, please read carefully the instructions provided at every question. The study will take about 10 minutes to complete. **All completed questionnaires will be anonymised for analysis and only group data will be reported.** The results will be published in the form of conference papers and journal publications in the future.

We would like to receive your replies before 31 May 2014.

If you require any further information about the study please contact one of the researchers listed below.

Dr. Daisy Volmer +3727375298 daisy.volmer@ut.ee

Prof. Jyrki Heinämäki +3727375286 jyrki.heinamaki@ut.ee

Thank you for participation in the study!

**Questionnaire to academic staff and pharmacy students**

Please follow the instructions provided for each question.

1. The professional knowledge about MD is important for pharmacists.

*Please select only one option!*

| What is this for? | Strongly Disagree | Disagree | Neither Agree nor Disagree/Undecided | Agree | Strongly Agree |
| --- | --- | --- | --- | --- | --- |
|  | 1 | 2 | 3 | 4 | 5 |

1. How would you rate the importance of MD knowledge?

*Please select only one option in each line!*

| Not important | Somewhat important | Moderately important | Important | Very important |
| --- | --- | --- | --- | --- |
| 1 | 2 | 3 | 4 | 5 |

- 1. In better understanding the role of MD in healthcare
  2. Provision of traditional community pharmacy services
  3. Provision of extended community pharmacy services
  4. Other..................... (please specify)

1. At my university, courses/lectures are provided on

*Please select only one option in each line!*

Yes No

- 1. MD general principles
  2. practical use of different types of MDs
  3. practical use of DDPs
  4. Other....................

1. If “Yes” for any of the above, please provide details on course(s) name(s) and the approximate number of hours. If there are no independent courses on MDs/DDPs, please name the course(s) and number of hours devoted to the topic in the course(s).

........................................................................................................................................................................................................................................................................................................................................................................................................................................................................................................................................................................................................................................................................................................................................................................................................................................................................................................................................................................................................................................................................................................................................................................................................

1. The courses/lectures on MDs are

*Please select only one option in each line!*

Yes No Not applicable (NA)

- 1. obligatory
  2. elective
  3. other.................. (please specify)

1. At my university, MD courses/lectures are taught by

*Please select only one option in each line!*

Yes No NA

- 1. professionals on the field of MD,
  2. university lecturers with medical or pharmacy education and without special education on MD,
  3. representatives of MD industry,
  4. practicing medical doctors or nurses
  5. Other........................ (please specify)

1. At my university, the faculty/department of pharmacy employs specialists with professional knowledge in the MD field?
   1. Yes
   2. No
   3. NA
2. If yes, please specify the field of expertise of employee and describe how the education is approached (general and/or practical):

............................................................................................................................................................................................................................................................................................................................................................................................................................................................................................................................................................................

1. Would you consider increasing the education on MD for pharmacy students in the future?

*Please select only one option!*

| Not At All | To a Small Degree | To a Moderate Degree | To a Considerable Degree | To a Great Degree |
| --- | --- | --- | --- | --- |
| 1 | 2 | 3 | 4 | 5 |

1. Would you consider it important to have international courses of the type „teaching the teachers” to expand MD knowledge among pharmacists?

*Please select only one option!*

| Not important | Somewhat important | Moderately important | Important | Very important |
| --- | --- | --- | --- | --- |
| 1 | 2 | 3 | 4 | 5 |

1. According to your knowledge, what institutions (in your country, in Europe) could provide teachers for this type of courses?

.............................................................................................................................................................................................................................................................................................................................................................................................................................................................................................................................................................................................................................................................................................................................................................................................................................................................................

1. Would you like to include some information or comments concerning MD teaching/studies at your university or more general comments concerning this topic?

.............................................................................................................................................................................................................................................................................................................................................................................................................................................................................................................................................................................................................................................................................................................................................................................................................................................................................

1. Demographic data

Country

University

Staff member (please specify position)………………………..

Student

Bachelor BSc, study year………….

Master MSc, study year…………...
