# Supplementary material for: Perception of the Professional Knowledge of and Education on the Medical Technology Products among the Pharmacists in the Baltic and Nordic Countries—A Cross-Sectional Exploratory Study
Source: Pharmacy (Basel). 2016 Oct 13;4(4):29. doi: 10.3390/pharmacy4040029 (PMC5419374; doi:10.3390/pharmacy4040029)
Supplement: Supplementary file 1 [file pharmacy-04-00029-s001.zip › pharmacy-148992-supplementary/pharmacy-148992-supplementary3.docx]

**Questionnaire to Pharmacy Professional Organisations**

Dear colleague,

The use of medical devices (MDs) and various drug-delivery products (DDPs) is rapidly growing in the health care. Community pharmacies are an important source for counselling and dispensing of MDs and DDPs.

Today there is a clear need for improvement of the knowledge and skills of pharmacists in MD counselling services exists. To date, however, very limited and fragmented information is available about education and awareness of both community and hospital pharmacists on MDs and DDPs.

**The aim of this study is to assess the content, quantity and potential future developments of educational activities on MDs and DDPs for pharmacy students and pharmacists in Nordic and Baltic countries**.

For a better understanding of the topic, the following definitions describe some aspects relating to medical devices in more detail:

*Medical device (MD)—*any instrument, apparatus, appliance, material or other article, whether used alone or in combination, including the software necessary for its proper application, intended by the manufacturer to be used for human beings for the purpose of:

- diagnosis, prevention, monitoring, treatment or alleviation of disease;
- diagnosis, monitoring, treatment, alleviation of or compensation for an injury or handicap;
- investigation, replacement or modification of the anatomy or of a physiological process;
- control of conception; and which does not achieve its principal intended action in or on the human body by pharmacological, immunological or metabolic means, but which may be assisted in its function by such means.

*MDs can be further classified as:*

*Personal medical device (PMD)—*not defined in the European medical device legislation. PMDs, as a major sub-group of MDs, could be described as portable, consumer-focused technologies that can be used at home for health and fitness trending, chronic disease management, and elderly patient monitoring (examples of PMDs: blood pressure monitors, glucometers, cholesterol meters, pregnancy/ovulation tests).

Or:

*Drug-delivery product (DDP)*—a device that is intended to administer a medicinal product within the meaning of the medicinal product directive (examples of DDPs: inhalation devices, insulin pens).

**We would like to invite you or your colleagues to complete the attached questionnaire.** The questionnaire is intended to be completed by members of professional organizations in community and hospital pharmacy dealing with practicing pharmacists and continuing education initiatives. We hope to receive one to two completed questionnaires from this type of organizations from all network countries.

Before completion of the questionnaire, please read carefully the instructions provided at every question. The study will take about 10 minutes to complete. **All completed questionnaires will be anonymised for analysis and only group data will be reported.** The results will be published in the form of conference papers and journal publications in the future.

We would like to receive your replies before 31 May 2014.

If you require any further information about the study please contact one of the researchers listed below.

Dr. Daisy Volmer +3727375298 daisy.volmer@ut.ee

Prof. Jyrki Heinämäki +3727375286 jyrki.heinamaki@ut.ee

Thank you for participation in the study!

**Questionnaire for professional organizations**

1. The professional knowledge about MD is important for pharmacists.

*Please select only one option!*

| Strongly Disagree | Disagree | Neither Agree nor Disagree/Undecided | Agree | Strongly Agree |
| --- | --- | --- | --- | --- |
| 1 | 2 | 3 | 4 | 5 |

1. How would you rate the importance of MD knowledge?

*Please select only one option in each line!*

| Not At All | To a Small Degree | To a Moderate Degree | To a Considerable Degree | To a Great Degree |
| --- | --- | --- | --- | --- |
| 1 | 2 | 3 | 4 | 5 |

- 1. In better understanding the role of MD in healthcare
  2. Provision of traditional community pharmacy services
  3. Provision of extended community pharmacy services
  4. Other...................... (please specify)

1. At my country, continuing education courses/lectures for practicing pharmacists are provided on

*Please select only one option in each line!*

Yes No

- 1. MT/MD general principles
  2. practical use of different types of MDs
  3. practical use of DDPs
  4. Other....................

1. If “Yes” for any of the above, please provide details on course(s) name(s) and the approximate number of hours. If there are no independent courses on MDs/DDPs, please name the course(s) and number of hours devoted to the topic in the course(s).

..........................................................................................................................................................................................................................................................................................................................................................................................................................................................................................................................................................................................................................................................................................................................................................................................................................................................................................................................................................................................................................................................................................................................................................................................................................................................................................................................................................................................................................................................................................................................

1. The continuing education courses/lectures on MDs are

*Please select only one option in each line!*

Yes No Not applicable (NA)

- 1. elective
  2. obligatory
  3. other....................... (please specify)

1. MD continuing education courses/lectures are taught by

*Please select only one option in each line!*

Yes No NA

- 1. professionals on the field of MT/MD,
  2. university lecturers with medical or

pharmacy education and without special

education on MT/MD,

- 1. representatives of MT/MD industry,
  2. practicing medical doctors or nurses
  3. Other......................... (please specify)

1. According to your knowledge in your country are there employed specialists at university pharmacy faculty/department or other institutions with professional knowledge in the MD field?
   1. Yes
   2. No
2. If yes, please specify the field of expertise of employee and describe how the education is approached (general and/or practical):

....................................................................................................................................................................................................................................................................................................................................................................................................................................................................................................................................................................................

1. Would you consider increasing the focus on MD for pharmacy students in the future?

*Please select only one option!*

| Not At All | To a Small Degree | To a Moderate Degree | To a Considerable Degree | To a Great Degree |
| --- | --- | --- | --- | --- |
| 1 | 2 | 3 | 4 | 5 |

1. Would you consider it important to have international courses of the type „teaching the teachers” to expand MD knowledge among pharmacists?

*Please select only one option!*

| Not important | Somewhat important | Moderately important | Important | Very important |
| --- | --- | --- | --- | --- |
| 1 | 2 | 3 | 4 | 5 |

1. According to your knowledge what institutions (in your country, in Europe) could provide teachers for this type of courses?

..............................................................................................................................................................................................................................................................................................................................................................................................................................................................................................................................................................................................................................................................................................................................................................................................................................................................................

1. Would you like to include some information or comments concerning MD teaching/studies at your university or more general comments concerning this topic?

................................................................................................................................................................................................................................................................................................................................................................................................................................................................................................................................................................................................................................................................................................................................................................................................................................................

13. Demographic data

Country

Name of the professional organization or

Professional organization dealing with community pharmacies/hospital pharmacies?

Respondent details (position in the organization)

If practicing pharmacist (pharmacy manager/head pharmacist/year of the employment)?
